# Improved Survival of Periviable Infants after Alteration of the Threshold of Viability by the Neonatal Resuscitation Program 2015

**DOI:** 10.3390/children8010023

**Published:** 2021-01-04

**Authors:** Yen-Ju Chen, Wen-Hao Yu, Li-Wen Chen, Chao-Ching Huang, Lin Kang, Hui-Shan Lin, Osuke Iwata, Shin Kato, Mohamed Hamed Hussein, Yung-Chieh Lin

**Affiliations:** 1 Department of Pediatrics, National Cheng Kung University Hospital, College of Medicine, National Cheng-Kung University, Tainan 70457, Taiwan; yensweet@gmail.com (Y.-J.C.); fieldof19@gmail.com (W.-H.Y.); muffychen@gmail.com (L.-W.C.); huangped@mail.ncku.edu.tw (C.-C.H.); 2 Department of Obstetrics and Gynecology, National Cheng Kung University Hospital, College of Medicine, National Cheng-Kung University, Tainan 70457, Taiwan; kanglin@mail.ncku.edu.tw; 3 Department of Nursing, College of Medicine, National Cheng-Kung University, Tainan 70457, Taiwan; javagip@gmail.com; 4 Department of Neonatology and Pediatrics, Nagoya City University Graduate School of Medical Science, Nagoya, Aichi 467-8601, Japan; o.iwata@med.nagoya-cu.ac.jp (O.I.); shink@med.nagoya-cu.ac.jp (S.K.); 5 Department of Neonatology, Center of Maternal, Fetal and Neonatal Medicine, Saitama Medical Center, Saitama Medical University, Kawagoe, Saitama 350-8550, Japan

**Keywords:** periviable infants, borderline viability, extremely preterm infants, management, neonatal resuscitation program, survival rate

## Abstract

Periviable infants (PIs) born at 22–25 weeks gestational age (wGA) have a variable survival rate (49.7–86.2%) among hospitals. One factor involved in this difference may be the definition of the threshold of viability. The American Academy of Pediatrics revised the neonatal resuscitation program in late 2015 (NRP 2015) and altered the threshold of viability from 23 to 22 wGA. The impact on the survival of PIs after the guideline alteration has seldom been discussed. Since 2016, the unit of this study has implemented the renewed guideline for PIs. We retrospectively reviewed and analyzed the survival and clinical variables of PIs before and after implementation of the guideline, which included a 10-year cohort in a single center in Taiwan. There were 168 PIs enrolled between 2010 and 2019 (Epoch-I, 2010–2015; Epoch-II, 2016–2019), after excluding those with congenital anomalies and parent-decided comfort care. Compared to those in Epoch-I, the PIs in Epoch-II had significantly higher odds ratios (2.602) (95% confidence interval: 1.170–5.789; *p* = 0.019) for survival. Younger gestational age, small size for gestational age, cesarean delivery, low blood pH at birth, and surfactant therapeutic treatment were found to be significant risk factors associated with the survival of PIs (*p* < 0.05 for each). The altered threshold of viability by NRP 2015 may impact the survival of PIs. However, long-term follow-up for surviving PI is required in the future.

## 1. Introduction

In recent years, researchers have become increasingly interested in the care of periviable infants [1,2,3]. The care of periviable infants continues to challenge state-of-the-art medicine. Periviable birth is defined as delivery happening from 20^0/7^ to 25^6/7^ weeks gestational age (wGA) [4,5,6]. In the past two decades, there have been reports of increased rates of survival of periviable infants born at 22–25 wGA from single centers or large cohorts in Taiwan, Japan, USA, France, Germany, and Australia [7,8,9,10,11,12,13,14]. However, the reported survival rate of periviable infants varied between reports (49.7–86.2%) [9,11,15]. In particular, in infants born at 22–23 wGA, the reported survival rate is from 8% to 70% for infants born at 22 wGA [9,15] and from 25% to 75.4% for infants born at 23 wGA [13,15].

The causes of these great differences in observed survival between reports deserve more research attention. An explanation might be care quality [16,17], but relatively little is understood about the effect of the definition of the threshold of viability. A documented threshold of viability is important, and aggressive treatment for periviable infants may result in better survival without severe complications [7,13]. Japan has protected fetuses of ≥22 wGA since 1991 [18], and intact survivors aged ≥ 22 wGA have been reported [19]. In Taiwan, however, pregnant women may legally terminate their pregnancy before 24 weeks of gestation [20], and resuscitation for infants born at 22–23 wGA depends on documented guidelines. For the survival of periviable infants, consent from parents for intervention is an important initial step.

In late 2015, the American Academy of Pediatrics issued a new version of the neonatal resuscitation program (NRP 2015) and extended the threshold of viability from 23 to 22 wGA [21,22]. After the new NRP 2015, prenatal counseling provided a clear option for families to rescue their babies born at 22 wGA. However, limited research has investigated the impact of NRP 2015 on the survival of periviable infants. Since the unit of this study implemented NRP 2015 in 2016, we sought to evaluate the effectiveness of NRP 2015 on periviable infants’ survival. We hypothesized that the implementation of NRP 2015 with the altered threshold of viability might influence the survival of periviable infants. We also investigated the effects of clinical variables on survival.

## 2. Materials and Methods

### 2.1. Study Design

This study was approved by the Institutional Review Board of National Cheng Kung University Hospital ER-98-135. The study aimed to identify the differences in the survival of resuscitated periviable infants between the two periods: before and after the implementation of NRP 2015. We retrospectively conducted applied comparative cohort research for two epochs. All periviable infants admitted to our unit from January 2010 until December 2019 were enrolled. The first epoch (Epoch-I) included years 2010–2015, and the second epoch (Epoch-II) included years 2016–2019.

The exclusion criteria were diagnosis with a major anatomical anomaly or chromosome anomaly or parental refusal for resuscitation. Infants with postnatal age of greater than one week at admission were also excluded.

The study was conducted in June 2020, when the enrolled infants had all been discharged. The survival outcomes and clinical variables of the two epochs were collected through the use of an electronic medical charting system.

### 2.2. Study Setting and Care Policies

This study was conducted in a 20-bed tertiary neonatal intensive care unit (NICU) at the National Cheng Kung University Hospital in Tainan, Taiwan. The care volume of this unit is approximately 350 neonates treated yearly, including approximately 80 very-low-birthweight infants. Two neonatologists, two residents, and one nurse practitioner are regularly in charge of the admitted infants.

#### 2.2.1. Antenatal Counseling

Antenatal counseling was provided for all parents who might have periviable infants. The threshold of viability was extended from 23 to 22 wGA in 2016. Counseling was conducted by a perinatal team. Repeated discussions could be held to address parents’ concerns. Three options were provided: full resuscitation (Plan A), palliative care (Plan B), and an intermediate plan (Plan C) of noninvasive respiratory support with nasal continuous positive airway pressure (CPAP), intravenous fluids, and no endotracheal intubation. Palliative care (Plan B) included drying of the periviable infant (PI) and suctioning of airways, if needed, with no other respiratory management. These infants were folded in warm, dry covers and given to their mothers to hold with their partners. For emergency precipitated preterm labor where counseling could not be held, Plan C was initially applied, and PIs were admitted to the NICU if resuscitation in the delivery room succeeded in keeping the heart rate (HR) above 100 and oxygen saturation above 85%. If a family hesitated in receiving Plan A or Plan B, Plan C encouraged families to let babies receive a trial of non-invasive positive ventilation in delivery rooms and to observe their infant’s progress in the NICU. Options for withdrawal care were also provided if severe sequelae related to poor neurological outcomes occurred after full resuscitation [7].

#### 2.2.2. Perinatal Management

Avoidance of hypothermia began in the perinatal period [21,22]. A heated nebulizer was used below plastic wrap to sustain the relative humidity during time-consuming procedures. The incubator humidity was controlled up to 85% for the first two weeks of postnatal life. Umbilical cord milking was performed by a pediatric resuscitating team [23]. Umbilical cord blood sampling from the cord close to the placenta was used for blood culture, a complete blood count (CBC) test, and a biochemical profile [24]. This method aimed to decrease blood loss and accelerated the application of antibiotics after birth.

#### 2.2.3. Respiratory Management

The core idea of respiratory care in this unit was to provide gentle respiratory support first. Respiratory support in the delivery room was initiated with continuous positive airway pressure (CPAP) and escalated based on the neonatal resuscitation program. Gentle respiratory support with the implementation of a saturation target range was our routine policy [25]. Surfactant was only used for therapeutic purposes, not for prophylaxis, where infants with respiratory distress (RDS) need 40% oxygen to reach PaO2 ≥ 80 mmHg in their arterial blood gas. Discussion about extubation readiness in intubated periviable infants was made daily by the attending physician in charge with all medical team members in order to minimize the number of mechanical ventilation days.

#### 2.2.4. Nutrition Management

Parenteral and enteral nutrition were initiated soon after birth. Enteral feeding started with the trophic mother’s own breast milk or human donor milk; this was maintained for 3 to 5 days and increased to 10–20 mL/kg/d if infants were able to tolerate it. Fortification began when the daily enteral feeding was providing more than 100 mL/kg/d [26].

#### 2.2.5. Infection Control and Other Management

Nonsterile gloves were used after hand hygiene before contact with the infants [27]. Invasive arterial blood pressure was measured by the peripheral artery line from birth to the second week of life if central arterial lines were not accessible [28]. Patent ductus arteriosus (PDA) treatment was conducted according to the guidelines [29,30]. Serial cranial ultrasonography was performed by senior pediatric neurologists on postnatal days 1, 3, 7, 14, and 30, with follow-up as indicated. Prophylaxis fluconazole was routinely given to all periviable infants until full feeding status was attained [31].

#### 2.2.6. Discharge Policy and Post-Discharge Follow-Up Program

The infants were discharged when they reached a postmenstrual age of at least 35 weeks and had a stable body temperature under ambient room temperature, adequate growth velocity, stable oxygen peripheral saturation in room air or under a low-flow nasal cannula, and no apneic episodes for one week. After discharge, all infants received fortification with their mother’s milk and monthly follow-ups with palivizumab injections to prevent respiratory syncytial virus infection. The neurodevelopmental follow-up program was arranged by a case manager for all very preterm infants [32,33].

### 2.3. The Primary Outcome and Variable Definitions

In this study, the primary outcome was survival at discharge. The survival rate calculations included NICU-admitted PIs initially assigned to plans A and C without including the PIs in plan B (initially assigned to palliative care). For this study, we determined the survival outcomes at discharge and the chronological ages and postmenstrual ages of the infants at discharge. The first day after birth was defined as postnatal Day 1. The clinical variables included sex, gestational age, and bodyweight at birth (BBW). The z score of BBW was calculated using the Fenton preterm growth chart, and small for gestational age was defined as a BBW for wGA of less than the 10th percentile [34].

Maternal pregnancy-related complications were observed from obstetric charts. Culture-proven sepsis occurring within three days after birth was defined as early-onset sepsis. Hypothermia was defined as a first body temperature obtained at the admission of less than 36.5 °C. The blood gas pH was determined from the first sample obtained within 4 h of life in the NICU. The therapeutic surfactant therapy for respiratory distress syndrome (RDS) was recorded, regardless of the status of intubation.

### 2.4. Statistics Analysis

Data analysis was performed in June 2020. This study was observational and, therefore, the study population was not recruited on the basis of a statistical power calculation. All analyses were performed using SPSS (Version 26, IBM, Armonk, NY, USA). The dependence of outcomes on the clinical variable was assessed by adjusting for a priori covariates chosen to account for the clinical relevance and collinearity between variables. Multivariate logistic regression analysis was performed to evaluate the crude effects of the potential independent variables on the outcome. Continuous variables were compared using the Mann-Whitney *U* test, whereas categorical data were compared using the chi-square test or Fisher’s exact test, where applicable. A *p*-value of less than 0.05 was considered to indicate a significant difference.

## 3. Results

**Figure 1:**
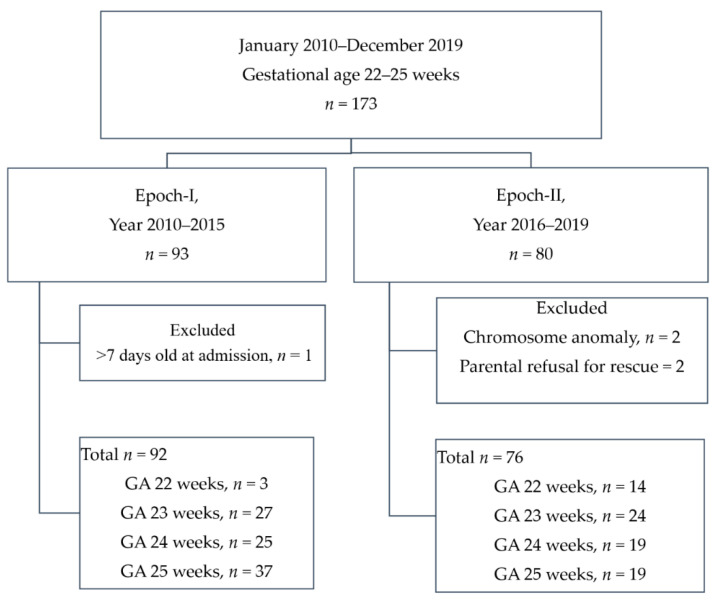
A flow chart showing the enrollment scheme. GA: gestational age.

This study enrolled 168 periviable infants in the 10-year cohort for final analysis, as shown in Figure 1. A total of five infants were excluded: two infants who received parent-decided palliative care, two infants with trisomy 21 and trisomy 18, and one infant who was referred to the unit for treatment of retinopathy at 138 days of life. Detailed data of all PIs born or transferred to the unit in which the study was conducted are provided in Supplemental Table S1.

Epoch-I had 92 periviable infants over a six-year period, an average of 15.3 periviable infants per year, and Epoch-II had 76 periviable infants over a four-year period, an average of 19 periviable infants per year. There was an annual increment of 18% in the number of infants being treated. The majority of this increment (33%) was accounted for by periviable infants born at a GA of 22 weeks (Epoch-I vs. Epoch-II: 3 in six years vs. 14 in four years, 7-fold increase) and those born at GA 23 weeks (Epoch-I vs. Epoch-II: 27 in six years vs. 24 in four years, 1.3-fold increase).

The perinatal and early clinical characteristics of the 168 periviable infants born in this 10-year cohort are listed in Table 1. At discharge, the overall survival rate was 61.3%. The periviable infants in Epoch-II showed younger gestational age (*p* = 0.002), lower birth bodyweight (*p* <0.001), and more frequent early-onset sepsis (*p* = 0.038) than those in Epoch-I. Maternal gestational morbidities did not differ between the two epochs.

Table 1 shows that 86.2% of cases resulting in mortality occurred in the first month of life, and the median age of death was four days of life. Hence, the link between perinatal and early life factors and survival is further explored in Table 2. Late complications of prematurity, such as retinopathy relating to prematurity, chronic lung disease, and late-onset sepsis, are not within the scope of this article.

**Figure 2:**
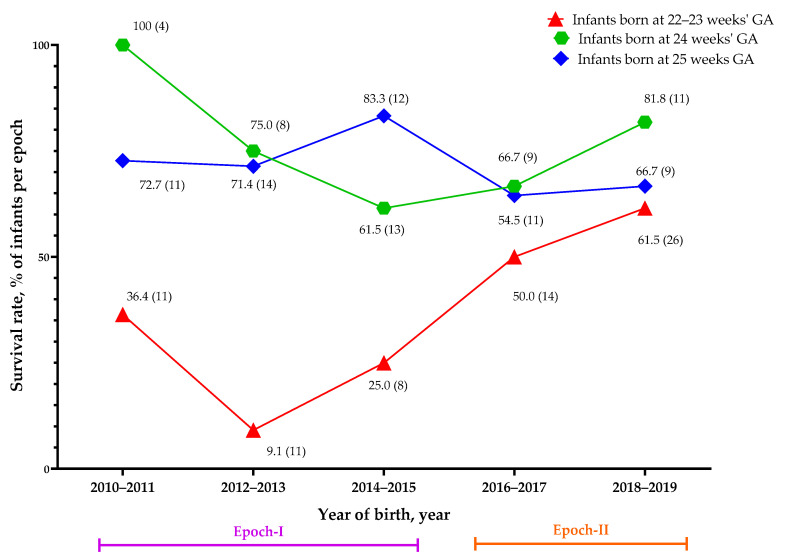
The biennial survival rate in each gestational age group. The data, along with the symbols, represent the survival rate as a percentage (total numbers in each population). GA: gestational age.

We further analyzed the biennial trend for the survival rate by gestational age, as shown in Figure 2. After the implementation of NRP 2015, Figure 2 shows that infants born at a gestational age of 22–23 weeks increased in patient number and survival through years 2016 to 2019, while the number and survival rate of infants born at 24 or 25 weeks of gestation were stable. In 2018–2019, infants born at a gestational age of 22–23 weeks had similar survival rates (61.5%) as infants born at 24 wGA (66.7%).

Content analysis using logistic univariate analysis was undertaken to determine which variables were associated with survival. Table 2 presents the results of this analysis.

The unadjusted correlation between survival and the epoch was found to be positive (odds ratio (OR) 1.415) but statistically insignificant (*p* = 0.279). Survival was observed to be significantly positively correlated with increased gestational age, larger BBW, higher Apgar score at 5 min, and higher blood pH at birth (*p* = 0.002, <0.001, 0.001, and 0.005, respectively). Survival correlated negatively with the occurrence of small for gestational age (SGA), preeclampsia, and surfactant-treated respiratory distress syndrome (RDS) (*p* = 0.014 and <0.001, respectively).

To investigate the effect of the changed guidelines on survival, we performed a multivariate analysis (Table 3). The dependence of survival on multiple clinical variables was assessed. Covariates accounting for clinical relevance and collinearity a priori were chosen from Table 2 for the multivariate analysis. The significant or important clinical variables were found to be epoch of birth, gestational age, sex, SGA, antenatal steroid therapy, maternal pre-eclampsia, birth by cesarean section, hypothermia, blood pH at admission, and therapeutic surfactant therapy in RDS.

Table 3 shows that periviable infants in Epoch-II had a 2.602 odds ratio of survival compared with those born in Epoch-I, and this was shown to be significant (*p* = 0.019) by the multivariate analysis model (Table 3). In this model, favorable factors of survival included a later gestational age (OR 1.884, *p* = 0.003) and higher blood pH at birth (OR 1.94, *p* = 0.012), while the unfavorable factors of survival were SGA (OR 0.154, *p* = 0.004), cesarean section (OR 0.417, *p* = 0.033), and RDS requiring therapeutic surfactant therapy (OR 0.306, *p* =0.005).

There was an improvement in survival odds in GA 23 PIs in Epoch-II compared with those born in Epoch-I. There was no difference in the survival odds of GA 24 and GA 25 infants between epochs (Table 4).

## 4. Discussion

In this study, we aimed to report the improved survival rate for periviable infants in a single center after the implementation of NRP 2015. We retrospectively compared the survival rates of periviable infants born in two epochs treated with different guidelines. Overall, after the implementation of the new guidelines, the adjusted survival rate significantly increased. We thus report that the NRP 2015 has had a significant impact on adjusted survival in periviable infants. However, long-term follow-up of these vulnerable infants is indicated for those who survived.

### 4.1. Survival Rate of Periviable Infants

The survival rate of periviable infants varies greatly between studies and countries [7,8,9,10,11,12,13]. Although the related ethics issues are under debate, aggressive treatments improve the survival rate of periviable infants, especially those born at 22–23 weeks GA [7,9]. Ample reports have shown that the survival rate of periviable infants has increased [17,35,36]; however, these studies included periviable infants born before the altered NRP 2015. The work of Stoll et al. demonstrated survival rates among infants born at 22 wGA of 6% in 1993–1997 and 7% in 2008–2012 in the USA. However, the work of Ishii et al. showed a survival rate of 37.3% among infants born at 22 wGA in 2003–2005 in Japan. Existing research has focused on care quality but has failed to explore the threshold of viability defined by each law or set of guidelines.

To the best of our knowledge, no study has focused on the significance of the altered NRP 2015 on the survival of periviable infants. As reported in this study, 64.1% (50/78) of infants born at a GA of 22–25 weeks survived in 2016–2019. Despite that, periviable infants included in Epoch-II were of younger gestational age (*p* = 0.002), lower birth bodyweight (*p* <0.001), and more frequently suffered from early-onset sepsis (*p* = 0.038) than those in Epoch-I. This improvement in survival rate was probably caused by the change in guidelines. In this study, the population’s improved survival was observed to be significant in GA 22–23 infants (23.3% in Epoch-I vs. 60.5% in Epoch-II, *p* = 0.003, Fisher’s exact test). In Taiwan, population birth data show that 49.7% (359/723) of infants born at a GA of 22–25 weeks survived [15] and only 8% of infants born at 22 wGA survived when the NRP defined 23 wGA as the threshold for viability [15]. Taiwan should have a good opportunity to provide healthcare to every periviable infant admitted to the NICU [37], because Taiwan has national insurance, with a 99.8% coverage rate for the population for every live birth [38]. Hence, we believe that the altered threshold of viability is a good first step for the survival of periviable infants.

During this ten-year cohort, infants born at GA 24–25 weeks in the two epochs experienced different clinical management bundles but with a similar concept of full NICU therapy for infants reaching the limit of viability, as regulated by national laws. This management of these infants was not affected by the change in the limit of viability by the 2015 NRP. On the other hand, the management of infants born at GA 22–23 weeks in both epochs experienced a change in both the limit of viability as defined by the NRP and in the management of clinical bundles over time. The latter could have influenced survival outcomes, although there was a significant improvement in the odds of survival in infants born at GA 23 weeks and not in those born at GA 24–25 weeks between the two epochs. This illustrates that changes in the management of clinical bundles over these ten years had a minimal effect on PI survival between the two compared epochs (Table 4).

### 4.2. Law, Guidelines, and the Debate of Ethics

The rescue of periviable infants remains an ethical issue [39]. Having clearly defined guidelines on viability is ethically very important and is a gray zone that the law does not cover. Taiwanese pregnant women have the legal right to terminate their pregnancy before 24 weeks of gestation [20], but Japanese law has prohibited induced abortion after 22 weeks of pregnancy since 1991 [18]. Having documented guidelines on the threshold of viability is important for the initiation of resuscitation or discussion between medical staff and families [40]. In Taiwan, in particular, the accreditation of tertiary hospitals requires that 90% of medical staff in a unit be qualified in the neonatal resuscitation program [41]. We believe that the altered NRP 2015 and the requirement for trained medical staff are providing periviable infants with a greater chance of receiving aggressive care in Taiwan.

Sequelae among periviable survivors are always subject to unquestionable ethical concern. However, survival is the first step to living without disabilities or major morbidities. Our study supports the theory that periviable infants always need initial resuscitation to survive, but medical teams should consider quick withdrawal if efforts are not succeeding [18]. To date, considerable research supports the view that a higher number of periviable infants could survive without morbidities [7,13].

### 4.3. Risk or Potential Factors Related to the Survival of Periviable Infants

Besides the gestational age, many factors may affect the survival of periviable infants. Our findings provide strong evidence of statistically significant relationships between survival and clinical variables, such as cesarean section, blood pH at birth, and surfactant treatment after birth. In this study, antenatal steroid treatment was found to have a non-significant but positive correlation with survival (OR 1.892, *p* = 0.306); however, the rate at which antenatal steroids were given (87.5%) in this study was higher than that in other reports [42,43]. Our finding that the provision of antenatal steroids is linked with higher survival in periviable infants agrees with a number of recent studies [42,43,44]. The correlations between survival and blood pH at birth were significantly positive. Blood pH at birth was related to a low body temperature, hypotension, and hypoxia. The avoidance of perinatal acidosis events may be a critical point in improving survival. Our finding provides evidence that SGA is statistically significantly related to a lower OR for survival. Other studies also appear to support the notion that the occurrence of SGA has an impact on the survival of members of this population [45,46]. Contrary to our expectations, this study showed that being born by cesarean section results in a lower OR for the survival of periviable infants. Our study might have mixed emergency cesarean sections with elective cesarean sections. Few studies have investigated the effect of birth by cesarean section on the survival of periviable infants. Some evidence points to the benefits of cesarean section birth for periviable preterm infants [47,48]. However, the role of cesarean section birth in the survival of periviable infants deserves more research attention. In this cohort, RDS requiring therapeutic, not prophylactic, surfactant therapy, after adjusting for GA, was found to be a significantly unfavorable factor for survival. In Taiwan, the national insurance system only supports the therapeutic use of surfactant when infants need 40% oxygen to reach PaO_2_ ≥ 80 mmHg in their arterial blood gas. Surfactant therapy use has increased in periviable infants in recent years [17], but this risk factor emerging from our analysis revealed that periviable infants undergoing therapeutic, not prophylactic, surfactant therapy might need more research attention. Because the most common route of surfactant therapy is intubation, the need for intubation, not the need for surfactant, might be the risk factor associated with survival.

### 4.4. Limitations and Strengths

This study had some limitations. Firstly, in the studied population, being a single-center observational study resulted in a small sample size of periviable infants enrolled in the study, as well as the use of historical controls, would make it questionable accrediting change in practices and extending GAs limits of variability by the NRP 2015, to cause improvement in survival.

Secondly, studies concerned with survival outcomes of PIs have shown a number of variabilities in their inclusion and exclusion criteria. We calculated the survival rate without including PIs initially assigned to receive palliative care and confined our results to one epoch, a method that could be considered to limit the generalization of our results. A future multicentric retrospective study could be warranted.

## 5. Conclusions

The data presented here provide evidence that the alteration of the threshold of viability by the NRP 2015 might have helped to improve the survival rate of infants born at 22–25 weeks of gestational age. Although the survival rate of periviable infants was shown to be improved in this study, there is still much room for improvement, and future long-term follow-up investigation should be conducted.

## Figures and Tables

**Table 1:** Clinical characteristics of infants enrolled in this study.

	All Enrolled Infants	Epoch-I	Epoch-II	*p* Value
**Enrolled infants, *N***	168	92	76	
Sex, male	96 (57.1)	54 (58.7)	42 (55.3)	0.754
Gestational age, weeks	23.8 ± 1	24.0 ± 0.9	23.6 ± 1.0	**0.002**
Body weight at birth, grams	648 ± 122	681 ± 120	609 ± 112	**<0.001**
z score of body weight at birth	0.12 ± 0.98	0.01 ± 0.99	−0.29 ± 0.93	**0.048**
small for gestational age	18 (10.7)	6 (6.5)	12 (15.8)	0.078
**Maternal condition**				
Maternal age, years	31.6 ± 5.3	31.3 ± 5.0	31.9 ± 5.7	0.519
Multiple pregnancy	77 (45.8)	27 (29.3)	50 (65.8)	**<0.001**
Antenatal steroid therapy	147 (87.5)	78 (84.8)	69 (90.8)	0.349
Gestational diabetes mellitus	7 (4.2)	4 (4.3)	3 (3.9)	1.000
Maternal pre-eclampsia	23 (13.7)	9 (9.8)	14 (18.4)	0.119
PPROM	62 (36.9)	33 (35.9)	29 (38.2)	0.872
**Perinatal condition and treatment**				
Inborn	152 (90.5)	84 (91.3)	68 (89.5)	0.794
Cesarean section	82 (48.8)	45 (48.9)	37 (48.7)	1.000
Apgar score at 5 min	6 (5–8)	7 (5–8)	6 (5–7)	0.409
^1^ Body temperature at admission, °C	35.4 ± 1.2	35.4 ± 1.0	35.4 ± 1.3	0.869
Hypothermia 1 at admission	135 (80.4)	77 (83.7%)	58 (76.3)	0.248
Blood pH at admission	7.21 ± 0.15	7.22 ± 0.12	7.20 ± 0.17	0.512
Blood sugar at admission, mg/dL	93.3 ± 35.2	95.6 ± 35.7	90.4 ± 34.8	0.343
Early-onset sepsis	17 (10.1)	5 (5.5)	12 (16.0)	**0.038**
Surfactant-treated RDS	105 (62.5)	54 (58.7)	51 (67.1)	0.337
**Survival or mortality variable**				
Survived to discharge	103 (61.3)	53 (57.6)	50 (65.8)	0.340
PMA at discharge of survivor, weeks	41.5 ± 7.0	40.5 ± 8.0	42.7 ± 5.4	0.109
Mortality in the first month of life, *n* (% of all mortality cases)	56 (86.2)	34 (87.2)	22 (84.6)	1.000
Postnatal age of mortality, days	4.0 (2.5–18.5)	4.0 (3–19)	4.0 (2–11)	0.595

Values are number (%) or mean ± standard deviation or median (interquartile range) if not specifically mentioned. ^1^ body temperature < 36.5 °C; PPROM: preterm premature rupture of the membranes > 18 h; RDS: respiratory distress syndrome; PMA: postmenstrual age. Continuous variables were compared using the Mann-Whitney U test, whereas categorical data were compared using the chi-square test or Fisher’s exact test, where applicable. Statistical significance was assumed for *p* < 0.05 (indicated in bold).

**Table 2:** Dependence of survival at discharge on clinical variables: univariate analysis.

	OR	95% CI for OR		*p* Value
		Lower	Upper	
**Basic Information**				
Sex, male (ref. F)	0.916	0.488	1.717	0.784
Gestational age, Weeks	1.654	1.197	2.285	**0.002**
Birth BW, 100 g	1.691	1.269	2.252	**<0.001**
z score of birth BW	1.324	0.951	1.844	0.096
SGA (ref. no)	0.273	0.097	0.77	**0.014**
Epoch of birth, II (ref. I)	1.415	0.754	2.654	0.279
**Maternal condition**				
Maternal Age	1.01	0.953	1.071	0.735
Antenatal steroid (ref. no)	1.894	0.755	4.752	0.173
Antenatal MgSO4 (ref. no)	1.21	0.546	2.682	0.638
Gestational DM (ref. no)	1.607	0.303	8.538	0.578
Preeclampsia (ref. no)	0.349	0.141	0.861	**0.022**
PPROM (ref. no)	0.897	0.472	1.704	0.740
Chorioamnionitis (ref. no)	1.029	0.402	2.638	0.952
**Perinatal condition and treatment**				
Inborn (ref. no)	2.011	0.62	6.526	0.245
Cesarean section (ref. no)	0.587	0.314	1.099	0.096
Apgar at 5 min	1.309	1.113	1.539	**0.001**
Hypothermia (ref. no)	0.529	0.229	1.224	0.137
Blood pH, (ref. lowest level ^1^)	1.860	1.205	2.87	**0.005**
Blood sugar, mg/dL	0.997	0.988	1.006	0.485
Early onset sepsis (ref. no)	0.505	0.184	1.386	0.185
Therapeutic surfactant therapy (ref. no)	0.265	0.129	0.545	**<0.001**

^1^ Data were binned into four levels, ≤7.00, 7.01–7.16, 7.17–7.33, and ≥7.34. Ref., Reference; F: female; BW: body weight; SGA: small for gestational age; DM: diabetes mellitus; PPROM: preterm premature rupture of the membranes > 18 h; RDS: respiratory distress syndrome. OR: odds ratio, CI: confidence interval. Univariate logistic analysis was performed, and statistical significance was assumed for *p* < 0.05 (indicated in bold).

**Table 3:** Dependence of survival at discharge on clinical variables: multivariate analysis.

Covariates	OR	95% CI for OR		*p* Value	*R* Square
		Lower	Upper		0.348
Epoch					
II (2016–2019)	2.602	1.170	5.789	**0.019**	
I (2010–2015; reference)					
Antenatal steroid therapy					
Yes	2.137	0.676	6.759	0.196	
No (reference)					
Gestational age, weeks	1.884	1.244	2.852	**0.003**	
SGA					
Yes	0.154	0.044	0.541	**0.004**	
No (reference)					
Sex					
Female	0.953	0.438	2.075	0.904	
Male (reference)					
Cesarean section					
Yes	0.417	0.186	0.934	**0.033**	
No (reference)					
Hypothermia at admission					
Yes	0.518	0.19	1.414	0.199	
No (reference)					
Blood pH at birth (binned value) ^1^	1.94	1.156	3.255	**0.012**	
Therapeutic surfactant therapy					
Yes	0.306	0.133	0.705	**0.005**	
No (reference)					

OR: odds ratio; CI: confidence interval; RDS: respiratory distress syndrome; ^1^ data were binned into four levels: ≤7.00, 7.01–7.16, 7.17–7.33, and ≥7.34. A logistic regression with selected covariates was performed. Statistical significance was assumed at *p* < 0.05 (indicated in bold).

**Table 4:** Odds of survival of infants born in Epoch-II compared with Epoch-I in each gestational age group.

	Adjusted OR ^1^	95% C.I. for aOR		*p* Value
		Lower	Upper	
GA 25 W	1.314	0.275	6.285	0.732
GA 24 W	1.299	0.255	6.610	0.753
GA 23 W	10.314	2.430	43.774	**0.002**
GA 22 W	NA			

aOR: adjusted OR; C.I. Confidence interval; GA: gestational age, W: weeks, ^1^ adjusted with SGA, binned blood pH, and sex; NA: non-applicable because the logistic regression could not be performed due to the small number of infants in Epoch-I. A logistic regression with selected covariates was performed. Statistical significance was assumed at *p* < 0.05 (indicated in bold).

## Data Availability

Raw data are not made publicly available due to patient confidentiality.

## Supplementary Materials

The following are available online at https://www.mdpi.com/2227-9067/8/1/23/s1, Table S1: Details of periviable infants born or transferred to the study unit without congenital anomalies.
