# Supplementary material for: Improved Survival of Periviable Infants after Alteration of the Threshold of Viability by the Neonatal Resuscitation Program 2015
Source: Children (Basel). 2021 Jan 4;8(1):23. doi: 10.3390/children8010023 (PMC7824697; doi:10.3390/children8010023)
Supplement: Supplementary file 1 [file children-08-00023-s001.pdf]

Supplemental Table 1. Details of periviable infants born or transferred[*] to the study unit without congenital anomalies

Epoch	I		II	
Years	2010–2015		2016–2019	
Limitation of viability of gestational age in NRP, weeks	23		22	
Source of infants	Inborn	Out-born	Inborn	Outborn
Inborn in delivery room, $N_{22}/N_{23}/N_{24}/N_{25}$	3[a]/29/22/33	NA	17/24/17/14	NA
Resuscitated and died in delivery room, $N_{22}/N_{23}/N_{24}/N_{25}$	NA[a]/1/0/0	NA	0/0/0/0	NA
Palliative care and died in delivery room, $N_{22}/N_{23}/N_{24}/N_{25}$	NA[a]/2[b]/0/0	NA	2[c]/0/0/0	NA
Outborn, $N_{22}/N_{23}/N_{24}/N_{25}$	NA	0/1/3/4	NA	0/1/2/5
Admitted to the NICU alive and evaluated in the flowchart, $N_{22}/N_{23}/N_{24}/N_{25}$ (total N)	3/27/25/37 (92)		15/25/19/19 (78)	
Died after palliative care in the NICU, N_{22}/N_{23}	0/0		1[d]/1[d]	
Cases enrolled for analysis and received full NICU care, $N_{22}/N_{23}/N_{24}/N$25 (total N)	3/27/25/37 (92)		14/24/19/19 (76)	
Death after withdrawal treatment, $N_{22}/N_{23}/N_{24}/N_{25}$	0/0/0/0		1[e]/0/0/0	
Death after intensive treatment, $N_{22}/N_{23}/N_{24}/N_{25}$ (total N)	2/21/7/9 (39)		7/8/4/7 (26)	
Survival, $N_{22}/N_{23}/N_{24}/N_{25}$ (total N)	1/6/18/28 (53)		7/16/15/12 (50)	

NRP: neonatal resuscitation program; N_{number} : $N_{\text{gestational age groups}}$; NA: non-applicable; GA gestational age.

[a]. In Epoch I, neonatologists were not consulted for neonates born at 22 weeks of gestation unless the parents requested it.

[b]. One PI was assigned to receive palliative care during the antenatal consultation and another could not receive antenatal counselling and was assigned to palliative care after parental request during postnatal counselling.

[c]. One PI received palliative care following refusal of antenatal counselling, and another could not receive antenatal counselling and was assigned to palliative care after parental request during postnatal counselling.

[d]. Two PIs were emergently born due to maternal conditions and then admitted to the NICU with non-invasive respiratory support. The families soon chose palliative care for them after admission to the unit.

[e]. Withdrawal of treatment due to a severe intraventricular hemorrhage grade 4.

[*]. Transferred within 7 days of life.
